# Supplementary material for: Kendallknight: An R package for efficient implementation of Kendall’s correlation coefficient computation
Source: PLoS One. 2025 Jun 18;20(6):e0326090. doi: 10.1371/journal.pone.0326090 (PMC12176188; doi:10.1371/journal.pone.0326090)
Supplement: Attachment — Submitted filename: Review_to_Authors.docx [file pone.0326090.s001.docx]

**Kendallknight: An R Package for Efficient Implementation of Kendall’s Correlation Coefficient Computation**

**FIRST READ-THROUGH**

**Main goals:** to introduce the kendallknight R package to compute Kendall's correlation coefficient.

**Methods:** The study follows the computational approach proposed by Knight (1966) and later works. The authors focus on reducing computational complexity, enabling rapid calculations compared to traditional Base R implementations.

**Data:** Although not available, the data consist of large datasets typically used in econometric and statistical modeling.

**Main results:** according to the authors, the KendallKnight R package significantly reduces computation time. Furthermore, the package correctly handles edge cases and errors, ensuring reliability.

The work aligns with the scope of *Plos One*, as Plos One publishes research across multiple disciplines, including computational methods in statistics and econometrics.

The paper references foundational work by Knight (1966) and includes some recent computational advancements in Kendall's correlation.

The experimental design aligns with the study’s objectives.

The figures and tables are appropriate, but some of them would benefit from additional details.

The findings are interesting and relevant for researchers needing fast and accurate correlation computations. The conclusion aligns with the results but could be expanded to discuss broader applications in different fields.

The paper is helpful for statisticians and data scientists; however, the practical implications for non-experts should also be highlighted. A section on real-world applications would improve accessibility.

The English language is appropriate and understandable.

**DETAILED REVIEW**

**TITLE**

The title accurately reflects the manuscript's core focus.

**KEYWORDS**

The manuscript does not include keywords. To improve the discoverability of the research. Thus, it should be considered the inclusion of relevant keywords (e.g., Kendall’s correlation, efficient computation, R package, etc.)

**ABSTRACT**

The abstract outlines the study’s goals, methods, and main findings.

**INTRODUCTION**

The Introduction states the problem (the inefficiency of Kendall’s correlation coefficient computation in R for large datasets) and explains the computational complexity of the current implementation. It also introduces the proposed solution. However, there is **no mention of specific real-world applications** or case studies where this efficiency gain is critical. Some references to prior works are included, but their limitations are not thoroughly discussed. Thus, this section should be improved by:

(i) expanding on the practical implications of the referred efficiency gain (e.g., in econometrics, machine learning, finance);
(ii) Comparing briefly with other methods beyond R’s base implementation;
(iii) Clarify the novelty of this approach—how does it improve upon previous works?
(iv) In line 12, where it is written “O(nlog(n))”, it should be written “O(nln(n))”

**DEFINITIONS**

This section provides a mathematical definition of Kendall’s correlation coefficient. The notation and formulas are generally well-explained. However, there is no discussion of alternative correlation measures (e.g., Spearman’s correlation) and when Kendall’s would be preferable. Thus, compare Kendall’s coefficient with other non-parametric correlation measures.

**IMPLEMENTATION**

This section discusses the computational complexity and the transition from O(n²) to O(n log n).

The use of C++ for efficiency is mentioned, but the explanation lacks details on how the algorithm achieves this improvement. Thus, it should be improved.

Furthermore, the description is somewhat vague regarding the actual implementation steps.

Finally, no pseudocode or diagrams are provided, and it must be done.

**USAGE**

This section also requires some improvements:

(i) Provide concrete usage examples with real datasets.
(ii) Offer comparisons with Base R and other packages.
(iii) The code should be improved to allow for different confidence levels (other than 95%). Explain why it cannot be changed, or improve the code.
(iv) Table 2 – provide an explanation of the values (percentages) in the table.

**BENCHMARKS**

Benchmarks are included, which is a strong point of the paper. However, the details on the hardware and testing conditions seem superficial. It is unclear if different dataset sizes and structures (e.g., presence of many ties) were tested. Please:

(i) Specify the testing environment (CPU, RAM, OS, R version).
(ii) Show comparisons against multiple alternatives.
(iii) Test performance under various dataset conditions (e.g., small vs. large datasets, presence of ties).
(iv) Provide more details on how the percentages in Table 3 were obtained.
(v) On line 170 (page 7), check if “Table 2” is correct.
(vi) On line 173 (page 8), check if “Table 3” is correct.

**CONCLUSION**

This section effectively summarizes the paper’s contributions. However, it does not discuss limitations or potential areas for future research. Furthermore, there is no explicit discussion on reproducibility (e.g., availability of the code, package versioning). Thus, please clearly state the limitations, suggest future directions (e.g., further optimizations, extensions to other correlation measures), and include a statement on where to find the package and its documentation.

**References**

The head of this section is repeated. Correct it, please.
