# Supplementary material for: Kendallknight: An R package for efficient implementation of Kendall’s correlation coefficient computation
Source: PLoS One. 2025 Jun 18;20(6):e0326090. doi: 10.1371/journal.pone.0326090 (PMC12176188; doi:10.1371/journal.pone.0326090)
Supplement: Attachment — Submitted filename: review-comments.docx [file pone.0326090.s002.docx]

Dear Reviewers,

Thanks for the comments. I consider each of them as I considered these improve the draft.

I have re-written the article based on the received feedback.

However, I do not have a track changes feature in Quarto/Latex. I added a new rendered PDF.

**DATA**

Although not available, the data consist of large datasets typically used in econometric and statistical modeling.

*- My bad, I just cited the source (the dataset is available free of charge)*

**INTRODUCTION**

The Introduction states the problem (the inefficiency of Kendall’s correlation coefficient computation in R for large datasets) and explains the computational complexity of the current implementation. It also introduces the proposed solution. However, there is **no mention of specific real-world applications** or case studies where this efficiency gain is critical. Some references to prior works are included, but their limitations are not thoroughly discussed. Thus, this section should be improved by:

(i) expanding on the practical implications of the referred efficiency gain (e.g., in econometrics, machine learning, finance);
(ii) Comparing briefly with other methods beyond R’s base implementation;
(iii) Clarify the novelty of this approach—how does it improve upon previous works?
(iv) **In line 12, where it is written “O(nlog(n))”, it should be written “O(nln(n))”**

*- Each of these has been addresses now in a new introduction*

**DEFINITIONS**

There is no discussion of alternative correlation measures (e.g., Spearman’s correlation) and when Kendall’s would be preferable.

*- I added some additional details about this and a new section with an expanded detail.*

**IMPLEMENTATION**

This section discusses the computational complexity and the transition from O(n²) to O(n log n).

The use of C++ for efficiency is mentioned, but the explanation lacks details on how the algorithm achieves this improvement. Thus, it should be improved.

Furthermore, the description is somewhat vague regarding the actual implementation steps.

Finally, no pseudocode or diagrams are provided, and it must be done.

*- This section has now been largely expanded with pseudocode and a detailed description with a Tikz diagram*

**USAGE**

This section also requires some improvements:

(i) Provide concrete usage examples with real datasets.
(ii) Offer comparisons with Base R and other packages.
(iii) The code should be improved to allow for different confidence levels (other than 95%). Explain why it cannot be changed, or improve the code.
(iv) Table 2 – provide an explanation of the values (percentages) in the table.

*- The written section features: a new example, comparisons with base R and the Kendall package, and a different confidence level (80%). Indeed, in the memory allocation I forgot to divide by 1,000,000. I removed the %s as these are not really informative. The speed numbers are correct.*

**CONCLUSION**

This section effectively summarizes the paper’s contributions. However, it does not discuss limitations or potential areas for future research. Furthermore, there is no explicit discussion on reproducibility (e.g., availability of the code, package versioning). Thus, please clearly state the limitations, suggest future directions (e.g., further optimizations, extensions to other correlation measures), and include a statement on where to find the package and its documentation.

*- The conclusion was expanded to cover these points.*

**REFERENCES**

The head of this section is repeated. Correct it, please.

*- Fixed*
